# A rare cause of esophageal stenosis: Compression due to a thoracic osteophyte

**DOI:** 10.1002/deo2.260

**Published:** 2023-07-03

**Authors:** Suguru Miida, Yoshihisa Arao, Nobutaka Takeda, Shu Goto, Yuichi Kojima, Naruhiro Kimura, Kazunao Hayashi, Atsunori Tsuchiya, Shuji Terai

**Affiliations:** ^1^ Division of Gastroenterology and Hepatology Graduate School of Medical and Dental Sciences, Niigata University Niigata Japan; ^2^ Department of Gastroenterology and Hepatology Uonuma Institute of Community Medicine, Niigata University Medical and Dental Hospital Niigata Japan

**Keywords:** endoscopy, endosonography, esophageal perforation, esophageal stenosis, osteophyte

## Abstract

Several cases of esophageal stenosis caused by cervical vertebral osteophytes have been reported; however, few reports of esophageal stenosis caused by thoracic osteophytes are available. We describe the case of an 86‐year‐old man with esophageal stenosis caused by a thoracic osteophyte near the tracheal bifurcation. An endoscopic ultrasonography examination was scheduled to determine the cause of acute pancreatitis; however, lacerations observed at the bifurcation following endoscope removal during prior esophagogastroduodenoscopy led us to cancel the ultrasonography to avoid potential esophageal perforation. A review of the present case and six similar previous cases of thoracic osteophyte‐associated esophageal stenosis (identified via a systematic search of the PubMed database) demonstrated the clinical importance of a thoracic osteophyte near physiological esophageal stenosis. Esophagogastroduodenoscopy and computed tomography should be performed to screen for vertebral osteophytes before endoscopic ultrasonography, endoscopic retrograde cholangiopancreatography, and transesophageal echocardiography to avoid iatrogenic accidents.

## INTRODUCTION

Benign esophageal stenosis is commonly encountered in clinical practice. Stenosis is rarely attributed to extrinsic compressions, such as esophageal compression secondary to vertebral osteophytes. However, there are relatively more reports on esophageal stenosis secondary to cervical vertebral osteophytes^1^, ^2^ than on those secondary to thoracic osteophytes.

Esophageal stenosis is a risk factor for esophageal perforation. Mortality following esophageal perforation caused by transesophageal echocardiography in a patient with esophageal stenosis secondary to thoracic osteophytes has been reported previously^3^; thus, this condition should be treated cautiously.

Herein, we present a case of esophageal stenosis detected on esophagogastroduodenoscopy (EGD) prior to endoscopic ultrasonography (EUS) that was scheduled for evaluating the cause of pancreatitis. Computed tomography (CT) revealed benign esophageal stenosis secondary to a thoracic osteophyte near the tracheal bifurcation—a physiological esophageal stenosis site.

## CASE REPORT

An 86‐year‐old man was transferred to our hospital with suspected acute pancreatitis. Three months before his transfer, he was admitted to another hospital for right‐sided pneumonia. He had a fever (37.5°C), but his other vital signs were stable. Physical examination was unremarkable except for mild tenderness from the epigastric region to the lower abdomen.

Blood tests showed elevated serum hepatobiliary enzymes, pancreatic enzymes, and inflammatory response (Table 1). Dynamic contrast‐enhanced CT revealed acute pancreatitis. Cholelithiasis and choledocholithiasis were not noted; however, we considered the possibility of gallstone pancreatitis based on the blood test results. Because the pain was not severe, we decided against performing an urgent endoscopic procedure. We administered fluids, proteinase inhibitors, and intravenous meropenem. The following day, abdominal pain disappeared, and pancreatitis was treated conservatively with good progress. Magnetic resonance cholangiopancreatography further showed no common bile duct or gallbladder stones. The hepatobiliary enzyme levels normalized over time. We scheduled a EUS evaluation of the biliary system on hospitalization day 9.

**TABLE 1:** Laboratory test results on admission.

Hematology		
Reference range		
WBC (/μl)	11,050	3300–8600
RBC (×10^6^/μl)	4.92	4.35–5.55
Hb (g/dl)	13.8	13.7–16.8
PLT (/μl)	95,000	158,000–348,000

**Coagulation**		
Reference range		
PT ratio (%)	73	70–130
INR	1.2	0.9–1.1
APTT (s)	38.2	26.9–40.9
Fbg (mg/dl)	362	200–400
FDP (μg/ml)	6.8	<5.0

Biochemistry		
Reference range		
TP (g/dl)	6.4	6.6–8.1
Alb (g/dl)	3.1	4.1–5.1
BUN (mg/dl)	12	8–20
Cre (mg/dl)	0.60	0.65–1.07
T‐Bil (mg/dl)	2.8	0.4–1.5
D‐Bil (mg/dl)	1.2	<0.2
AST (U/L)	159	13–30
ALT (U/L)	60	10–42
LDH (U/L)	279	124–222
ALP (U/L)	149	38–113
γ‐GTP (U/L)	65	13–64
P‐Amy (U/L)	813	16–52
Lipase (U/L)	1,211	9–59
CRP (mg/dl)	1.8	≤0.14

Arterial blood gas (room air)		
Reference range		
pH	7.45	7.35–7.45
PCO_2_ (mmHg)	36	35–48
PO_2_ (mmHg)	72	83–108
HCO_3_^−^ (mmol/L)	24	22–26
BE (mmol/L)	0.3	‐2.0–2.0
Lac (mmol/L)	1.8	1.8–1.9

**Tumor markers**		
**Reference range**		
CEA (ng/ml)	3.5	<5.8
CA19‐9 (U/ml)	20	<37
DUPAN‐2 (U/ml)	<25	≤150

Abbreviations: Alb, albumin; ALP, alkaline phosphatase; ALT, alanine aminotransferase; APTT, activated partial thromboplastin time; AST, aspartate aminotransferase; BE, base excess; BUN, blood urea nitrogen; CA19‐9, carbohydrate antigen 19‐9; CEA, carcinoembryonic antigen; Cre, creatinine; CRP, C‐reactive protein; D‐Bil, direct bilirubin; DUPAN‐2, duke pancreatic monoclonal antigen type 2; Fbg, fibrinogen; FDP, fibrin degradation products; Hb, hemoglobin; HCO_3_^−^, bicarbonate ion; Lac, lactate; LDH, lactate dehydrogenase; P‐Amy, pancreatic amylase; PCO_2_, partial pressure of arterial carbon dioxide; PLT, platelet; PO_2_, partial pressure of arterial oxygen; PT, prothrombin time; INR, international normalized ratio of prothrombin time; RBC, red blood cell; T‐Bil, total bilirubin; TP, total protein; WBC, white blood cell; γ‐GTP, gamma‐glutamyl transpeptidase.

**FIGURE 1:**
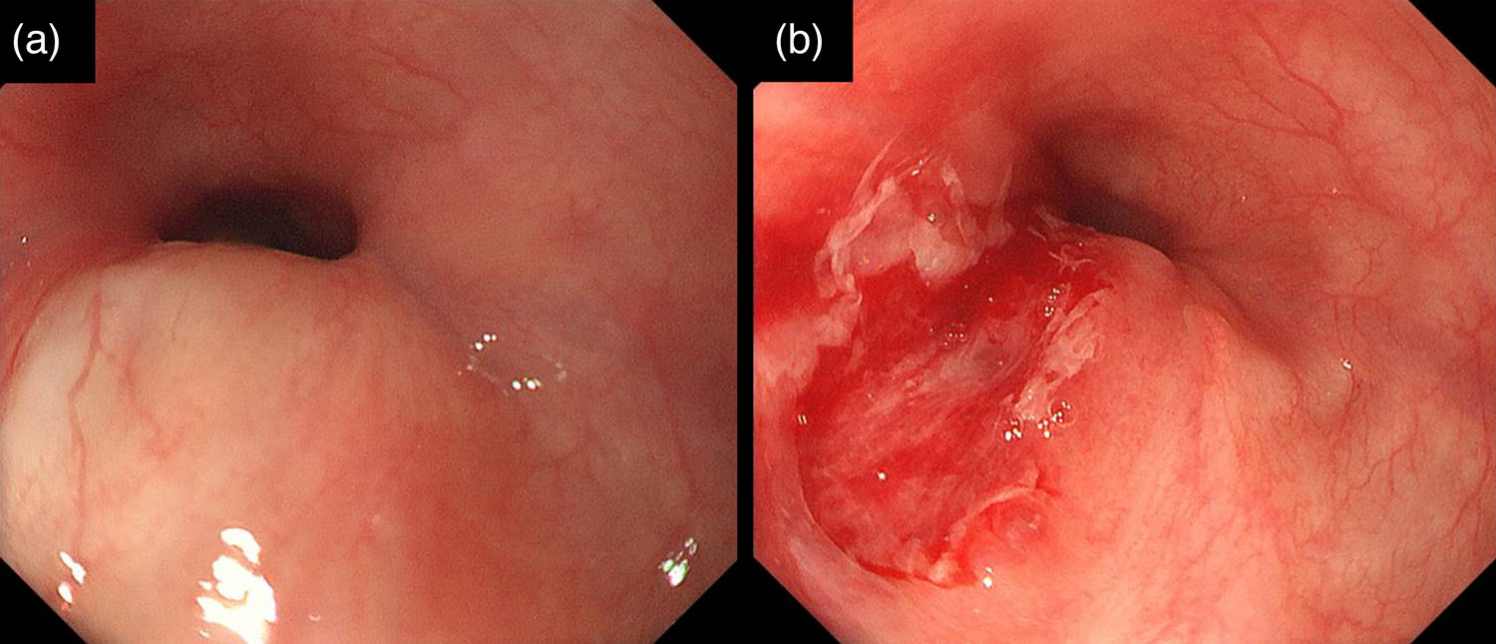
Endoscopic appearance of the esophagus. (a) Endoscope insertion: Severe stenosis due to extramural compression of the posterior wall is identified in the middle thoracic esophagus. (b) Endoscope removal: A laceration is observed at the stenosis.

**FIGURE 2:**
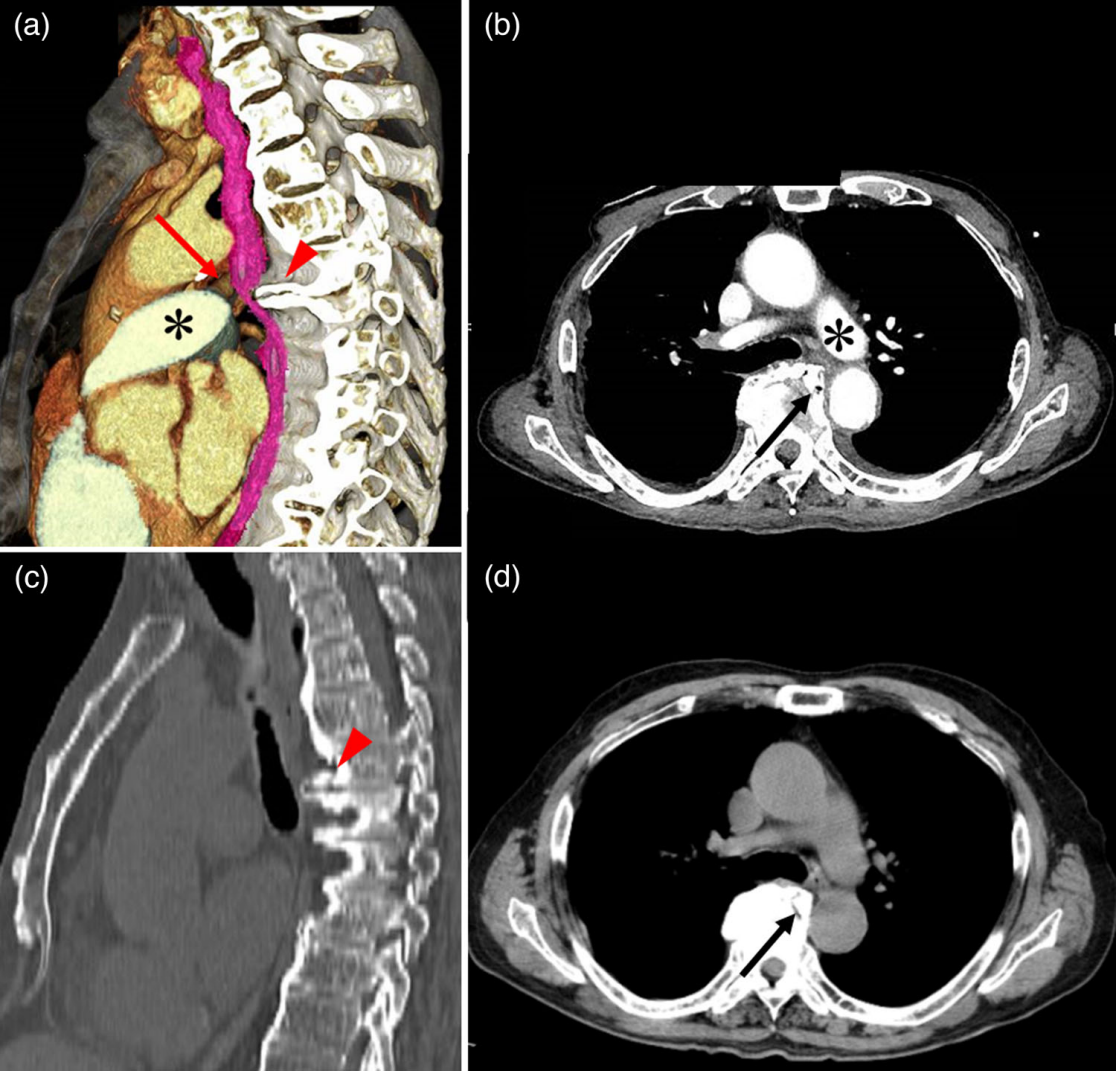
Computed tomography (CT) image on admission (a) Volume‐rendered, reconstructed sagittal section of the contrast‐enhanced CT image: The esophagus (red arrow) is constricted due to extrinsic compression from an osteophyte (red arrowhead) on the sixth thoracic vertebra, and the anterior esophageal surface is restricted by the left pulmonary artery (*). (b) Axial section of the contrast‐enhanced CT image: The esophagus has migrated to the left side of the trachea due to compression from the vertebral osteophyte (black arrow). The migration of its anterior surface is restricted by the left pulmonary artery (*). (c) Sagittal section of a plain CT image obtained at another hospital 8 years ago: The vertebral osteophyte (red arrowhead) appears smaller in size. (d) Axial section of the plain CT image obtained from another hospital 8 years ago: The vertebral osteophyte (black arrow) appears smaller in size.

The patient's first EGD (GIF‐H290Z; Olympus, Tokyo, Japan) was performed for screening prior to EUS evaluation, revealing a narrowing of the lumen due to compression of the posterior wall in the middle thoracic esophagus (Figure 1a). The compressed area was gently elevated with no epithelial changes; it was very rigid and immobile. The endoscope passed through the stenosis with resistance, and a laceration was observed at the site during removal (Figure 1b). Thus, we decided against performing EUS owing to the risk of esophageal perforation due to the use of a large side‐view endoscope. Retrospective evaluation of volume‐rendered reconstructed sagittal CT sections obtained at admission revealed an esophageal constriction due to extrinsic compression by an osteophyte at the sixth thoracic vertebra (T6). The left pulmonary artery restricted the anterior esophageal surface (Figure 2a). Axial CT revealed that the esophagus had migrated to the left side of the trachea due to compression from the vertebral osteophyte (Figure 2b). The degree of osteophyte formation had intensified following a plain CT performed at another hospital 8 years ago (Figure 2c,d). The esophageal stenosis was near the tracheal bifurcation. Thus, we diagnosed the patient with esophageal stenosis secondary to extrinsic compression from a thoracic vertebral osteophyte. Because the symptoms were resolved by day 2, the patient was monitored without additional treatment; he resumed eating on day 10 and was discharged on day 17 (Figure S1). Written informed consent was obtained from the patient to publish this case report and accompanying images.

## DISCUSSION

In this case, a thoracic vertebral osteophyte near the tracheal bifurcation (a physiological stenosis) led to benign esophageal stenosis. Extrinsic compression is a relatively rare cause of esophageal stenosis. There have been few reports of enlarged mediastinal and hilar lymph nodes secondary to infection and abnormalities in the right subclavian artery and other blood vessels.^4^, ^5^ Moreover, vertebral osteophytes are known to cause esophageal stenoses, and there are numerous reports of aspiration and dysphagia caused by cervical osteophytes.^1^, ^2^ Conversely, there are few reports on esophageal stenoses caused by thoracic osteophytes. Indeed, the cricoid cartilage in the cervical spine prevents forward movement of the esophagus, and stenosis is more likely to occur around the cervical spine. The thoracic esophagus is a relatively mobile structure that can be displaced anteriorly or laterally without compression; thus, stenosis is rare in this region. However, the esophagus is vulnerable to extrinsic compression at the tracheal bifurcation (near T4–T5) and diaphragmatic hiatus (near T9–T10) because the anatomical space between the esophagus and vertebral column is notably reduced.^6^ These sites are known as physiological stenosis sites in the esophagus.

We performed a literature search of the PubMed database using the following search strings: thoracic AND osteophyte; thoracic AND spondylosis. Six cases were described in the six case reports identified. Table 2 summarizes the clinical information of these cases and the present case. In all cases, patients were aged >60 years and involved men. We hypothesized that this was because of the higher prevalence in men. The incidence of vertebral body osteophytes is 50% in women and >80% in men >50 years of age.^7^ Symptoms of dysphagia accounted for more than half of the cases (four cases). The diagnosis was mainly based on CT and surgical findings. The stenosis was near the diaphragmatic hiatus in four cases and near the tracheal bifurcation in one case (i.e., our case), consistent with the physiological sites of stenoses in the esophagus. Two of these progressed to perforation and emergency surgery. Case 1 (Table 2) is of a patient who developed an esophageal perforation after transesophageal echocardiography during transcatheter aortic valve replacement for severe symptomatic aortic stenosis.^3^ The patient died despite undergoing surgery for esophageal perforation.

**TABLE 2:** Summary of the seven cases of esophageal stenosis secondary to thoracic vertebral osteophytes identified to date.

Case	Authors	Year of publication	Age (years)	Sex	Chief complaint	Endoscopy	Diagnosis modality	Location	Treatment	Outcome
1	Royer et al.^3^	2020	79	M	None (esophageal perforation during TEE)	+	CT Intraoperative findings	T7–8	Surgery	Death
2	Cai et al.^11^	2003	73	M	Dysphagia	‐	CT Barium swallow	T9–10	PPI	Improvement
3	Kilincalp et al.^12^	2015	76	M	Dysphagia	+	CT	T9–10	Diet modification and anti‐reflux and swallowing therapy	Improvement
4	Underberg‐Davis et al.^13^	1991	76	M	Postprandial chest tightness	+	Chest radiography Barium swallow	T9–10	Accidental improvement of esophageal food impaction during barium examination	Improvement
5	Rathinam et al.^14^	2010	63	M	Dysphagia (esophageal perforation after esophageal bougie dilation)	+	Water‐soluble contrast challenge Intraoperative findings	Around EGJ	Surgery	Improvement
6	Rana et al.^15^	2012	65	M	Dysphagia	+	CT barium swallow EUS	N/A	N/A	N/A
Present case	Present case	2023	86	M	None	+	CT	T6	Observation	No change

Abbreviations: CT, computed tomography; EGJ, esophagogastric junction; EUS, endoscopic ultrasonography; M, male; PPI, proton pump inhibitor; TEE, transesophageal echocardiography.

In the present case, because EGD was performed before EUS, we avoided perforation. Iatrogenic injuries are the most common cause of esophageal perforation, with an associated mortality rate of 13.2%.^8^ From 2002 to 2013, the use of upper EUS increased yearly across all age groups in the United States.^9^ This trend is expected to have continued to the present day worldwide. This may be owing to the establishment of EUS‐guided fine‐needle aspiration, interventional EUS techniques, and the aging population. Vertebral osteophytes are common imaging findings in 20%–30% of older adults, and their number and size increase with age.^10^ Although no specific treatment guidelines have been established, we suggest that EGD be performed prior to endoscopic procedures involving the use of side‐view endoscopes (such as EUS or endoscopic retrograde cholangiopancreatography) and transesophageal echocardiography to reduce iatrogenic accidents. Patients with planned EUS or endoscopic retrograde cholangiopancreatograph may have already undergone an abdominal CT; however, a chest CT is also crucial to evaluate the presence of thoracic osteophytes.

Reflecting on the necessity of treatment for esophageal stenosis in this case, surgery was considered radical, but because the patient was elderly, the indication for treatment had to be carefully considered. Given his history of aspiration pneumonia, we could not rule out the possibility that esophageal stenosis was the trigger; however, during his hospitalization, he did not show any symptoms of esophageal stenosis or aspiration. Therefore, we did not treat the esophageal stenosis in this case. If symptoms of esophageal stenosis were to develop, in accordance with previous reports, we would first consider improving the diet and anti‐reflux and swallowing therapy and then consider surgery.

Our study had some limitations. During the literature review, we may have missed some cases of thoracic osteophyte‐associated esophageal stenosis in less symptomatic patients (as in our case). All the cases of esophageal stenosis secondary to thoracic osteophytes occurred in males; however, the number of cases analyzed was very small (*n* = 7). Vertebral body osteophytes are common findings in women and should be evaluated regardless of sex. Furthermore, we evaluated the stenosis morphologically using EGD and CT; fluoroscopic examination with a water‐soluble contrast agent or barium would have enabled a better morphological and functional evaluation.

In conclusion, we experienced a case of esophageal stenosis caused by a thoracic osteophyte near the tracheal bifurcation—a physiological stenosis site. When performing EUS, endoscopic retrograde cholangiopancreatograph, and transesophageal echocardiography, especially in older adults, prior screening for vertebral osteophytes using EGD and CT can reduce endoscopic iatrogenic accidents. As the esophagus is usually collapsed, it may be difficult to evaluate luminal stenosis by CT alone. We believe that the combination of CT and EGD can provide a more reliable diagnosis.

## CONFLICT OF INTEREST STATEMENT

None.

## Supporting information

Figure S1

References

## Data Availability

The data supporting this article will be shared on reasonable request to the corresponding author.
