# Supplementary figures and images for: A rare cause of esophageal stenosis: Compression due to a thoracic osteophyte
Source: DEN Open. 2023 Jul 3;4(1):e260. doi: 10.1002/deo2.260 (PMC10318124; doi:10.1002/deo2.260)

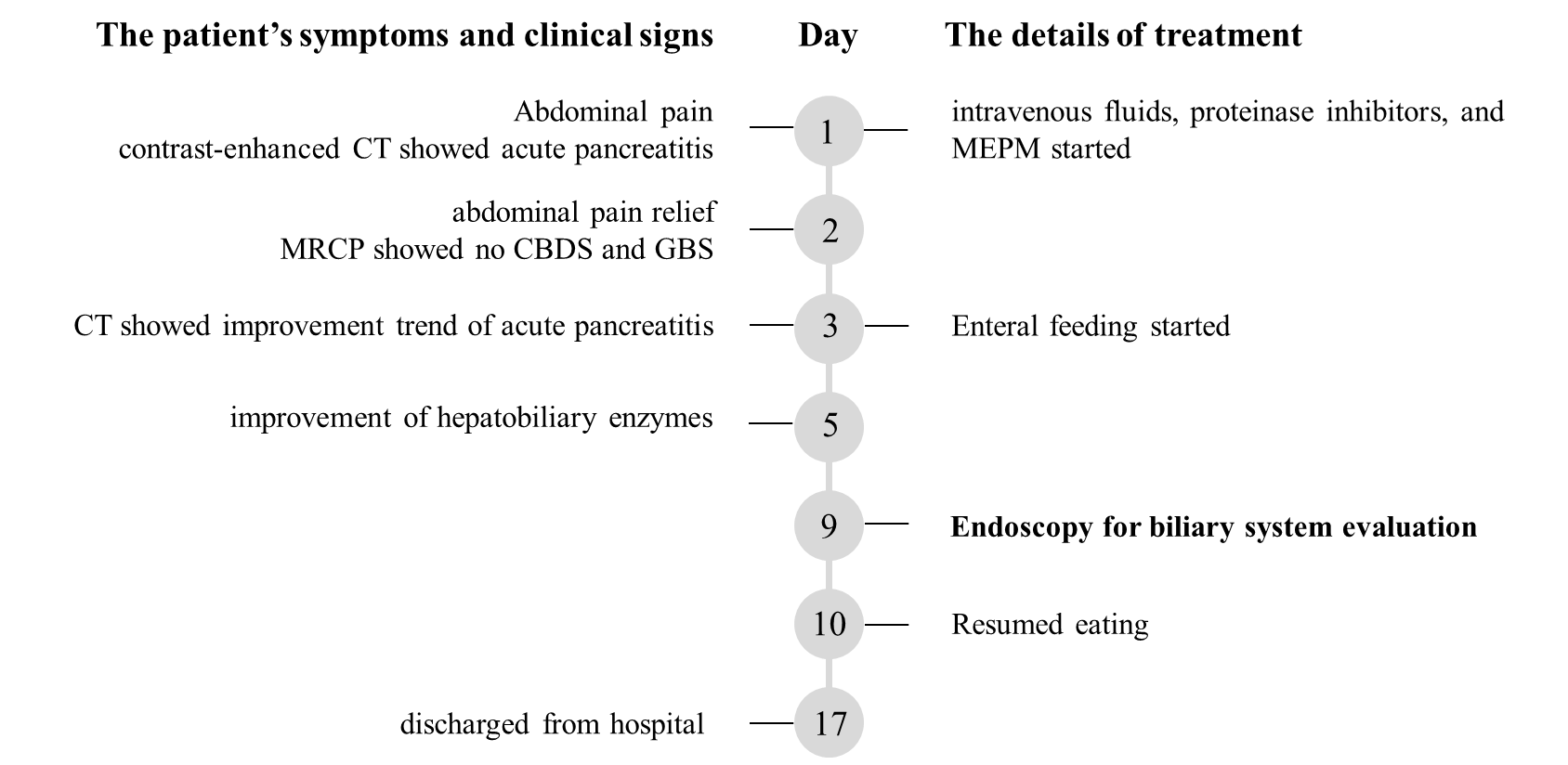

Supplement: Supplementary file 1 — Figure S1 [file DEO2-4-e260-s001.tiff]
