# Supplementary material for: A rare cause of esophageal stenosis: Compression due to a thoracic osteophyte
Source: DEN Open. 2023 Jul 3;4(1):e260. doi: 10.1002/deo2.260 (PMC10318124; doi:10.1002/deo2.260)
Supplement: Supplementary file 2 — References [file DEO2-4-e260-s002.docx]

**Supporting information**

***References***

11. Cai FZ, Rischmueller M, Pile K, Brady SJ. Dysphagia associated with lower thoracic spondylosis. *Rheumatology* *(Oxford)* 2003; 42: 1575–6.

12. Kilincalp S, Akıncı H, İsak ÖA, Çoban Ş, Yüksel İ. A rare cause of dysphagia: compression of esophagus by a giant thoracic spine osteophyte. *Endoscopy* 2015; 47: E1.

13. Underberg-Davis S, Levine MS. Giant thoracic osteophyte causing esophageal food impaction. *AJR Am J Roentgenol* 1991; 157: 319–20.

14. Rathinam S, Makarawo T, Norton R, Collins FJ. Thoracic osteophyte: Rare cause of esophageal perforation. *Dis Esophagus* 2010; 23: E5–8.

15. Rana SS, Bhasin DK, Rao C, Gupta R, Nagi B, Singh K. Thoracic spine osteophyte causing dysphagia. *Endoscopy* 2012; 44: E19–20.
